# Endovascular management of aortic aneurysm with severe neck angulation and/or iliac artery tortuosity using multiple stiff wire technique: a case series

**DOI:** 10.12688/f1000research.140435.2

**Published:** 2024-01-05

**Authors:** Taofan Taofan, Suko Adiarto, Iwan Dakota, Suci Indriani, Jonathan Edbert Afandy, Achmad Hafiedz Azis Kartamihardja, Sung-Gwon Kang, Renan Sukmawan

**Affiliations:** 1 Department of Cardiology and Vascular Medicine, Faculty of Medicine University of Indonesia, National Cardiovascular Center Harapan Kita, University of Indonesia Academic Hospital, Jakarta, Indonesia; 2 Assistant of Vascular Division, Department of Cardiology and Vascular Medicine, Faculty of Medicine University of Indonesia, National Cardiovascular Center Harapan Kita, University of Indonesia Academic Hospital, Jakarta, Indonesia, Jakarta, Indonesia; 3 Departement of Cardiology and Vascular Medicine, Faculty of Medicine Padjadjaran University, Hasan Sadikin General Hospital, Bandung, Indonesia; 4 Department of Radiology, Chosun University, Gwangju-Si, Gwangju-Si, South Korea

**Keywords:** TEVAR, EVAR, neck angulation, artery tortuosity, multiple stiff wire technique

## Abstract

**Background:**

Suitable aortic neck is one of the essential components for thoracic endovascular aortic repair (TEVAR) and endovascular aortic repair (EVAR). Advanced techniques were developed to adjust and compromise the aneurysm neck angulation but with adding additional devices and complexity to the procedure. We proposed a simple technique to modify severe neck angulation and/or iliac artery tortuosity by using the multiple stiff wire (MSW) technique.

**Method:**

Two femoral accesses were required for the MSW technique. A guidewire with a support catheter was inserted through the right and left femoral arteries and positioned in the abdominal or thoracic aorta. Wire exchanges were done with extra stiff wire in both femoral accesses. It can be considered to add multiple stiff wires to align the torturous neck / iliac artery. Delivery of the stent graft main body can be done via one of the accesses.

**Result:**

Six patients with different aortic pathology were admitted to our hospital. Four patients undergo EVAR procedure and two patients undergo TEVAR procedure. All patients had aortic neck angulation problems with one patient having iliac artery tortuosity. MSW technique was performed on the patients with good results. Follow-up CTA after 3 months revealed a good stent position without stent migration and no endoleak was found in all but one patient.

**Conclusion:**

MSW technique is a simple and effective technique to modify aortic neck/iliac artery angulation in TEVAR or EVAR procedure.

## Introduction

The endovascular aortic repair (EVAR) technique has been developed for over 30 years since the first report by Parodi, *et al*. ^1^ and recommended over open surgical repair by 2022 ACC/AHA Guideline for the Diagnosis and Management of Aortic Disease ^2^ in descending thoracic aortic aneurysm (TAA), ruptured descending TAA, abdominal aortic aneurysm (AAA), and ruptured AAA. A suitable aortic neck is one of the essential components for EVAR and hostile neck anatomy has been related to early graft failure and long-term adverse events. ^3^ To be categorized as hostile, an AAA neck must have any of the following criteria: (1) >2 mm reverse taper within 1 cm below the renal arteries, (2) ≥60° angulation within 3 cm below renal arteries, (3) ≤10 mm neck length, (4) ≥50% circumference of neck thrombus, and (5) >3 mm focal bulging in the neck. ^4^ Severe neck angulations as one of the hostile neck features appear to be linked with stent graft collapse, stent graft migration, type I endoleak, and late aneurysm rupture. ^5^

Nowadays, advanced techniques were developed to adjust and compromise the aneurysm neck angulation such as kilt technique which involves aortic-cuff stent-graft implantation before aortic main-body stent-graft implantation, and endostapling technique to reinforce device seal and fixation. ^6^^,^^7^ But, those techniques add additional devices and complexity to the procedure. Another concern also should be put in challenging iliac anatomy such as short length, small diameter, large diameter, and tortuosity since it has a significant role in the delivery system access site, stent graft sealing zone, pelvic flow source, and sometimes a combined iliac artery aneurysm that must be repaired. ^8^ We proposed a simple technique to modify severe neck angulation and/or iliac artery tortuosity by using the multiple stiff wire (MSW) technique with reports of application in six patients.

## Methods

The procedure was done at the National Cardiovascular Center Harapan Kita, Jakarta, Indonesia with around 50 EVAR and TEVAR cases per year. Vascular intervention consultant cardiologist with an experience more than 10 years done this procedure assisted by vascular intervention fellow student.

Two femoral accesses were required for the MSW technique. Then, a guidewire with a support catheter was inserted through the right and left femoral arteries and positioned in the abdominal or thoracic aorta. Wire exchanges were done with 0.035″ × 260 cm Lunderquist Extra Stiff Wire (Cook Aortic Interventions, Bloomington, USA) in both femoral accesses. It can be considered to add multiple stiff wires to align the torturous neck/iliac artery. Delivery of the stent graft main body can be done via one of the accesses. After the main body deployment, the contralateral extra stiff wire can be withdrawn from the access then contralateral limb extension cannulation and deployment can be done as required.

## Case presentation

### Case 1

**Figure 1.**
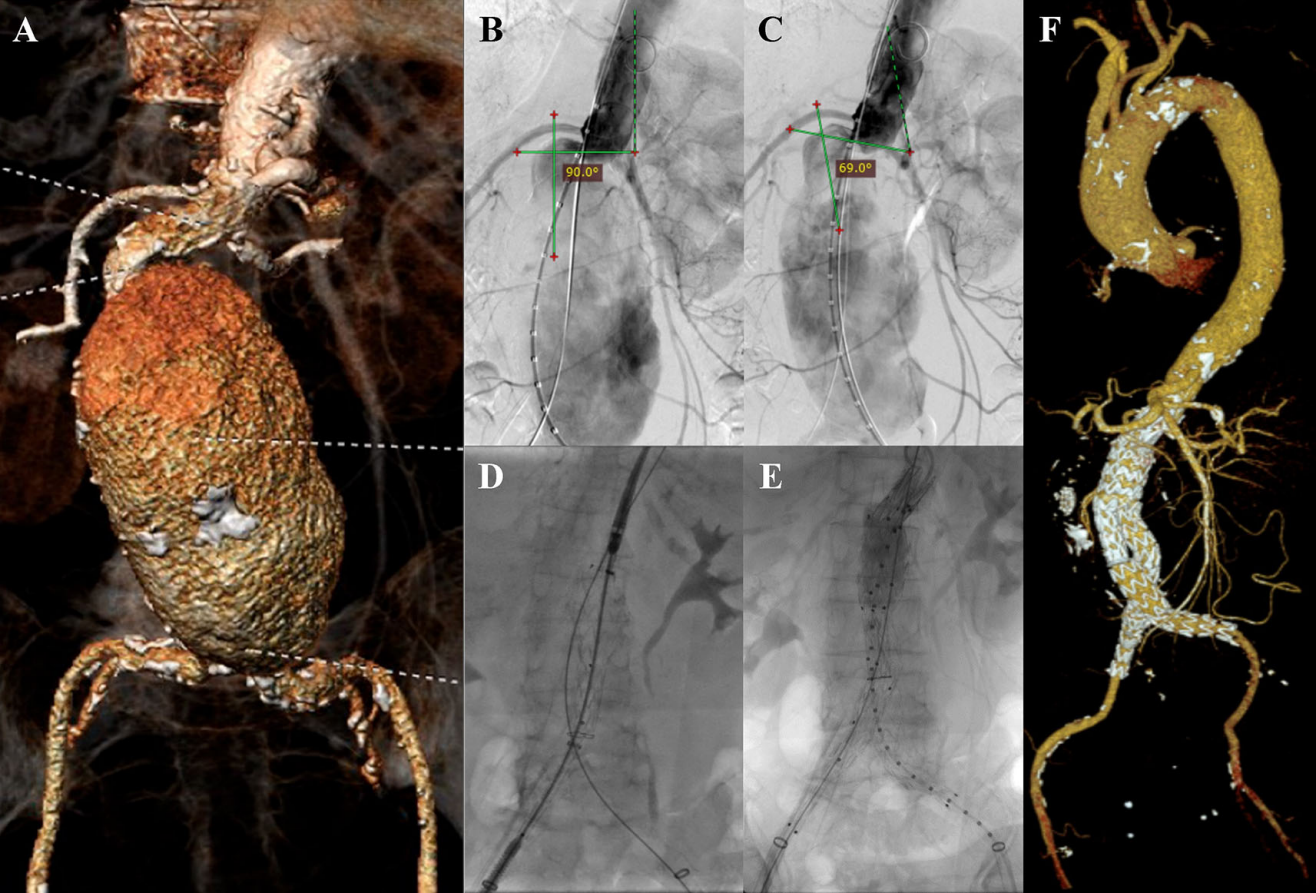
Endovacular aortic repair with multiple stiff wire technique of the 1 ^st^ patient. A. Preoperative volume rendering computed tomography angiography (CTA); B. Initial aortography showed 90° angulation of proximal neck; C. Aortography showed reduction of the proximal neck angulation after insertion of the second stiff wire into 69°; D. Stent graft main body deployment; E. Final aortography; F. Follow up volume rendering CTA after three months.

An 83-year-old woman was presented to the emergency department with abdominal pain that radiate into both lower limbs in the last hour. The patient had a history of hypertension and dyslipidemia. CT-Scan Angiography (CTA) revealed an abdominal aorta aneurysm from the inferior of superior mesentric artery until aortic bifurcation with length of 10.8 cm and maximum sac diameter of 7.4 cm and fusiform aneurysm of bilateral common iliac artery (CIA) with right diameter of 1.1 cm and left diameter of 1.5 cm. Infrarenal neck length is 22 mm with angulation of 90° ( Figure 1A). The patient then prepared for EVAR procedure.

The EVAR procedure was done with right and left femoral access that was gained with surgical cutdown technique. Initial aortography revealed a fusiform aneurysm at infrarenal abdominal aorta with short and tortuous neck with angulation angle of 90° ( Figure 1B). The MSW technique with two stiff wires was done to align the tortuous proximal neck. The proximal neck angulation was reduced into 69° ( Figure 1C). An Endurant II ETBF 23 mm × 13 mm × 145 mm stent graft main body (Medtronic, Santa Rosa, CA, USA) was deployed via right femoral artery (RFA) access ( Figure 1D). The extra stiff wire from left femoral artery (LFA) was withdrawn and canulation for contralateral limb extension with guide wire was done. An Endurant II ETLW 16 mm × 13 mm × 93 mm stent graft extension (Medtronic, Santa Rosa, CA, USA) was inserted and deployed overlapped with the main body from the neo bifurcation to the left CIA. The right Endurant II ETLW stent graft extension sized 16 mm × 13 mm × 93 mm (Medtronic, Santa Rosa, CA, USA) was deployed from the right femoral artery overlapped with the main body. Evaluation aortography revealed type IA endoleak and left CIA wasn’t filled completely. A stent graft balloon catheter was inserted from the LFA and few intrastent post dilatation was done at infrarenal abdominal aorta and proximal of the CIA. Another evaluation aortography was done and eventually, there still was type IA endoleak. It was decided to add another Endurant II ETCF 25 mm × 25 mm × 49 mm stent graft extension (Medtronic, Santa Rosa, CA, USA) proximal from the main body. Final aortography revealed a good position of the stent grafts, both renal and iliac arteries were filled with contrast, and no sign of endoleak was found ( Figure 1E). The total contras used was 120 mL Iopamidol 755 mg/mL, dose area product (DAP) 98.66 Gy.cm ^2^, and fluoro time was 41.21 minutes.

The patient was discharged without any complaints. The follow-up CTA after 3 months revealed stent expansion of 19.3 mm at infrarenal aorta (maximum diameter was 60.2 mm), good stent position without stent migration and no endoleak was found ( Figure 1F).

### Case 2

**Figure 2.**
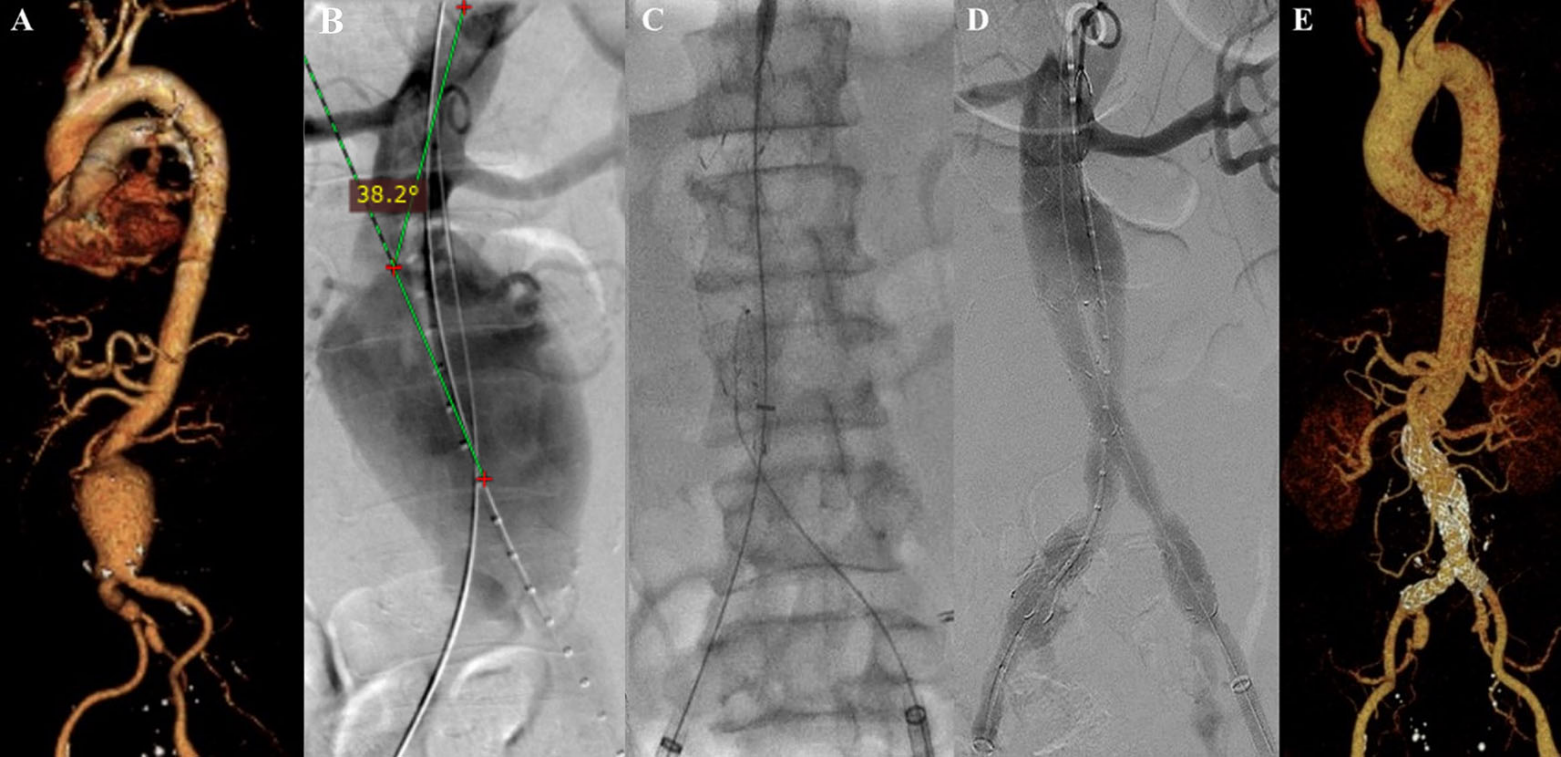
Endovacular aortic repair with multiple stiff wire technique of the 2 ^nd^ patient. A. Preoperative volume rendering computed tomography angiography (CTA); B. Initial aortography with two stiff wire showed straightening of the proximal neck angulation into 38.2°; C. Stent graft main body deployment; D. Final aortography; E. Follow up volume rendering CTA after three months.

A 55-year-old man presented to our hospital with stomach fullness and back pain in the past 2 weeks. The patient had a stomach massage before, but it didn’t relieve his symptoms. The patient denied history of hypertension and diabetes, but he was a smoker. CTA revealed a contained rupture of abdominal aortic aneurysm with intraluminal thrombus with a length of 10.32 cm and maximum sac diameter of 10.1 cm (maximum vascular diameter filled with contrast was 6.49 cm), Infrarenal neck length is 18.9 mm with angulation of 70.2° ( Figure 2A). The patient then prepared for EVAR procedure.

The EVAR procedure was done with puncture from right and left femoral access. Initial aortography revealed a contained rupture of aortic aneurysm at infrarenal abdominal aorta with enough length but tortuous neck. The MSW technique was applied to this patient and it reduced the proximal neck angulation into 38.2° ( Figure 2B). A SEAL Bifurcated Stent Graft sized 28 × 50 mm (S&G Biotech, Yongin, Korea) main body was deployed via RFA ( Figure 2C). The extra stiff wire from LFA was withdrawn and canulation for contralateral limb extension was done. SEAL Bifurcated Stent Graft extension 12(16) × 120 mm (S&G Biotech, Yongin, Korea) was inserted from left femoral artery access and deployed until the distal part was right above left internal iliac artery. The right stent graft extension with SEAL Bifurcated Stent Graft extension 12(16) × 120 mm (S&G Biotech, Yongin, Korea) was inserted from the RFA and deployed until the distal part was right above right internal iliac artery. Evaluation aortography revealed that contras filled the whole cover stent without endoleak, but the distal part of the right limb extension didn’t expand perfectly. After several post-dilatation was done with Reliant Stent Graft Balloon (Medtronic, Santa Rosa, CA, USA), the right limb extension expanded perfectly ( Figure 2D). The EVAR procedure was done without any complications. The total contras used was 30 mL Iopamidol 755 mg/mL, DAP 136.31 Gy.cm ^2^, and fluoro time was 33.49 minutes.

The patient was discharged without any complaints. Follow-up CTA after three months revealed stent expansion of 33.5 mm at infrarenal aorta (maximum diameter with thrombus was 69 mm), no stent migration, and no endoleak was found ( Figure 2E).

### Case 3

**Figure 3.**
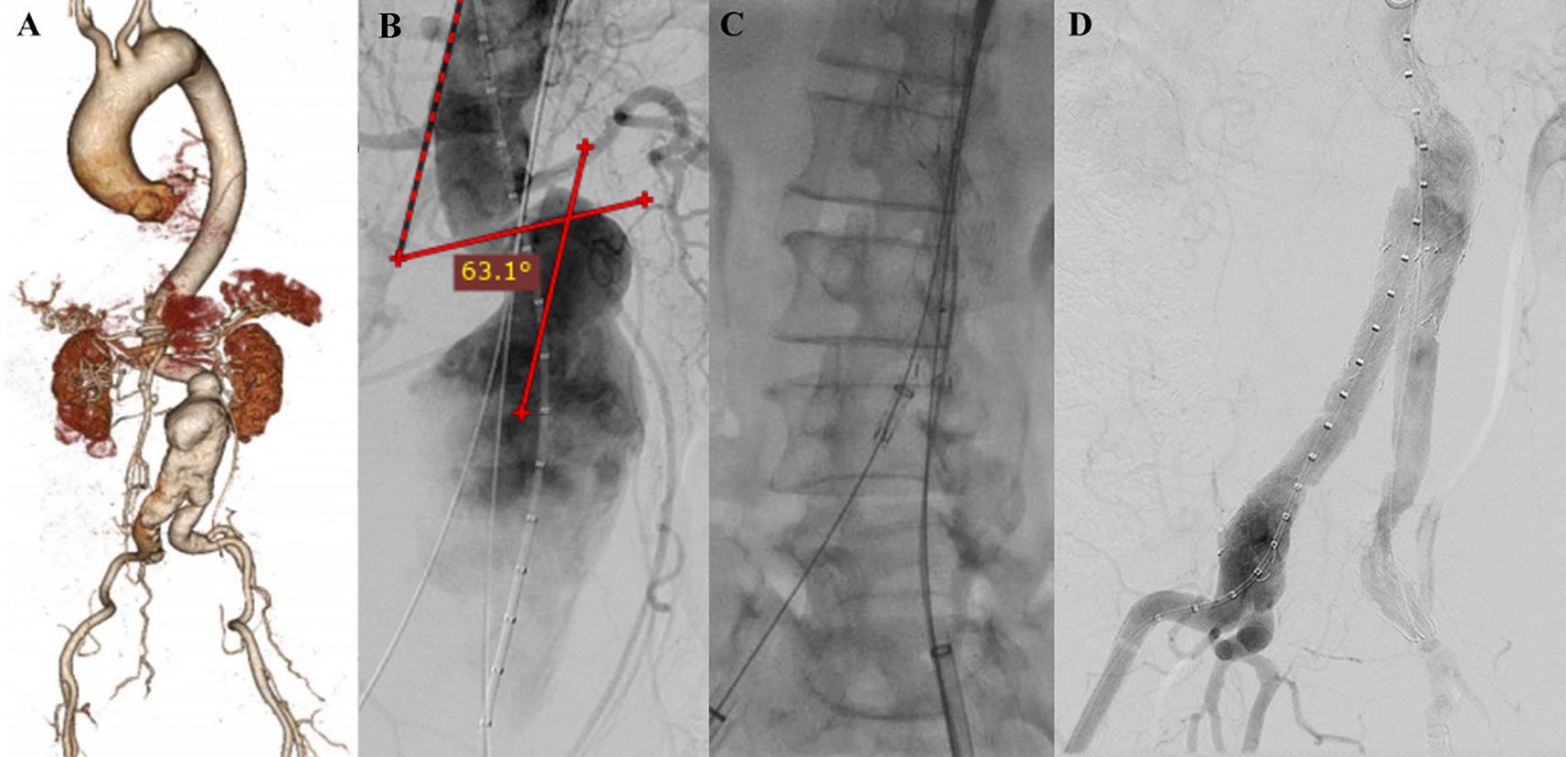
Endovacular aortic repair with multiple stiff wire technique of the 3 ^rd^ patient. A. Preoperative volume rendering computed tomography angiography (CTA); B. Initial aortography with two stiff wire showed straightening of the proximal neck angulation into 63.1°; C. Stent graft main body deployment; D. Final aortography.

A 69-year-old man presented to our hospital with stomach fullness accompanied by nausea and vomiting in the last three months. The patient also had a complaint of a palpable non-pain lump in his stomach. He denied history of hypertension or diabetes, but he was an ex-smoker. There was a palpable pulsatile mass in the abdominal region. CTA revealed dissection of abdominal aorta aneurysm with a length of 6.99 cm and maximum sac diameter of 5.95 cm (maximum vascular diameter filled with contrast was 3.89 cm), infrarenal neck length is 5.00 mm with angulation of 75.4° ( Figure 3A). The patient then prepared for EVAR procedure.

The EVAR procedure was done with puncture from right and left femoral access. The MSW technique was applied to this patient. Initial aortography revealed an aortic aneurysm at infrarenal abdominal aorta until bilateral CIA with enough length but tortuous neck. MSW technique reduced the proximal neck angulation into 63.1° ( Figure 3B). SEAL Bifurcated Stent Graft 24 × 50 mm main body (S&G Biotech, Yongin, Korea) was deployed via RFA ( Figure 3C). The extra stiff wire from LFA was withdrawn and canulation for contralateral limb extension was done. SEAL NOVUS Flared Limb Stent Graft extension 12(20) × 120 mm (S&G Biotech, Yongin, Korea) was inserted from LFA access and deployed until the distal part was right above left internal iliac artery. The right stent graft extension with SEAL NOVUS Flared Limb Stent Graft extension 12(20) × 100 mm (S&G Biotech, Yongin, Korea) was inserted from the RFA and deployed until the distal part was right above right internal iliac artery. Post-dilatation was done with balloon catheter in left CIA. Evaluation aortography revealed that contras filled the whole cover stent without endoleak ( Figure 3D). The EVAR procedure was done without any complications. The total contras used was 50 mL Iopamidol 612 mg/mL, DAP 59 Gy.cm ^2^, and fluoro time was 51.25 minutes.

The patient was discharged without any complaint. Unfortunately, the patient declined the evaluation CTA because of patient’s malignancy related condition that was diagnosed after the EVAR procedure, but he didn’t have any complaint related to the aortic or vascular disease after 3 months follow-up.

### Case 4

**Figure 4.**
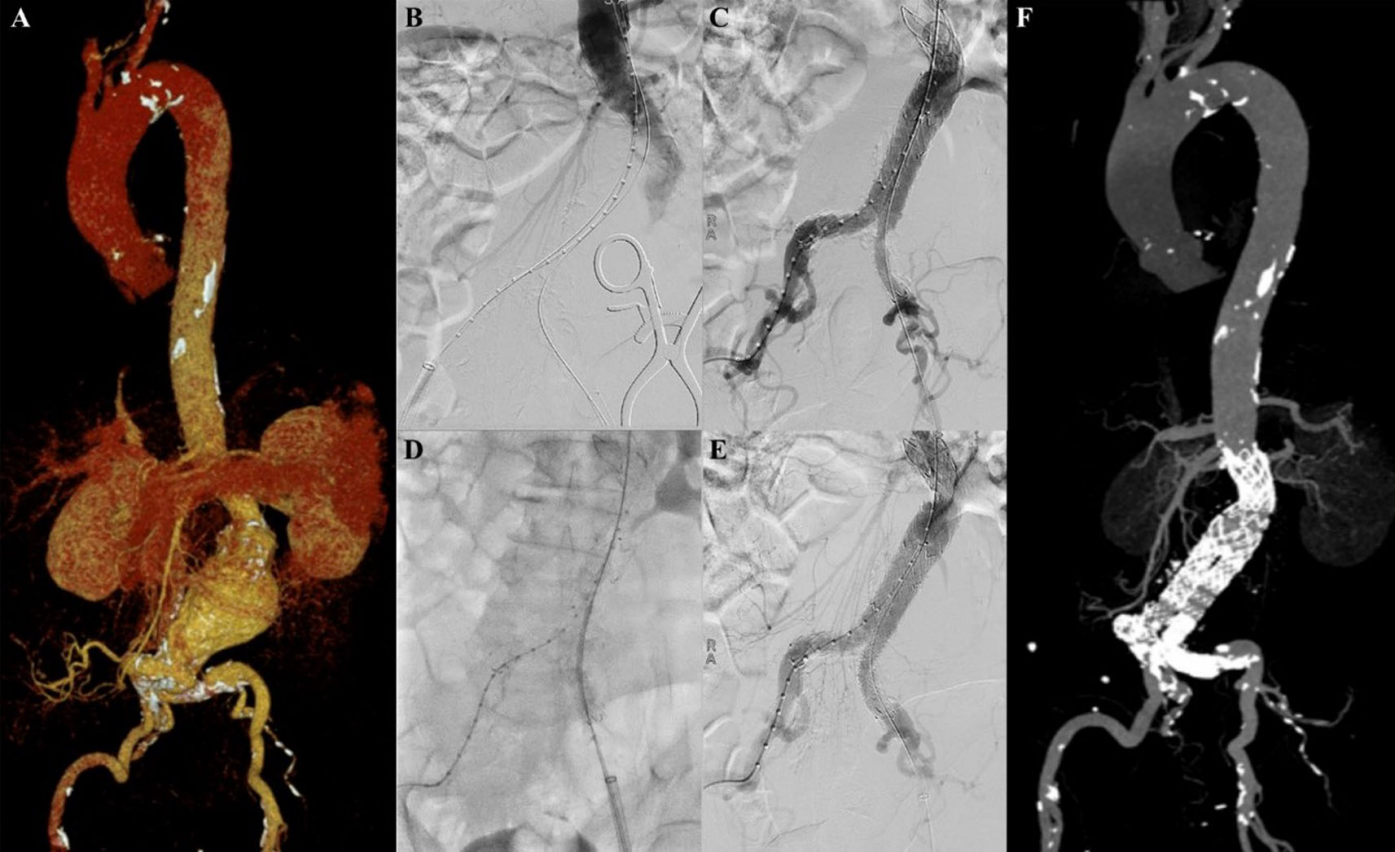
Endovacular aortic repair with multiple stiff wire technique of the 4 ^th^ patient. A. Preoperative volume rendering computed tomography angiography (CTA); B. Initial aortography; C. After stent implantation, arteriography revealed stenosis at left CIA; D. Iliac stent graft deployment; E. Final aortography; F. Follow up volume rendering CTA after three months.

A 71-year-old man was referred to our outpatient clinic with a pulsatile mass in lower left abdomen. The patient had a stable condition. CTA revealed impending rupture of fusiform abdominal aorta aneurysm with a length of 9.11 cm and maximum sac diameter of 6.3 cm (maximum vascular diameter filled with contrast was 3.63 cm), infrarenal neck length is 2.27 mm with angulation of 50°. The access site was tortuous and heavily calcified ( Figure 4A). The patient was prepared for elective EVAR and percutaneous transluminal angioplasty.

The EVAR procedure was done with right and left femoral access that was gained with surgical cutdown technique. Initial aortography revealed a fusiform aneurysm at infrarenal abdominal aorta with stenosis at both CIAs ( Figure 4B). Several plain balloon dilatations were done at both CIAs to facilitate the delivery of the stent graft main body. SEAL Novus stent graft main body sized 24 mm × 50 mm (S&G Biotech, Yongin, Korea) were deployed with SEAL bifurcated stent graft extension 12(18) × 100 mm (S&G Biotech, Yongin, Korea) until left CIA and SEAL bifurcated stent graft extension 12(16) × 100 mm (S&G Biotech, Yongin, Korea) until right CIA. Arteriography revealed there was still stenosis at left CIA ( Figure 4C). It was decided to add another stent at left CIA, but there was dificulty to pass the lesion. MSW technique was used to align the left CIA using Radiofocus Extra Stiff Wire 0.035″ × 260 mm (Terumo, Somerset, NJ). Dynamic 10 × 56 mm stent graft (Biotronik, Berlin, Germany) was implanted at left CIA ( Figure 4D). Final aortography revealed a good position of the stent-grafts, both renal arteries and iliac arteries were filled with contrast, and no sign of endoleak was found ( Figure 4E). The total contras used was 50 mL Iopamidol 755 mg/mL, DAP 59 Gy.cm ^2^, and fluoro time was 51.25 minutes.

The patient was discharged without any complaint. Follow-up CTA after 3 months revealed stent expansion of 23.0 mm at suprarenal aorta, 30.8 mm at infrarenal aorta (maximum diameter with thrombus was 59.1 mm), 28.8 mm at aortic bifurcation, 9.3 mm at right CIA, and 9.9 mm at left CIA, no stent migration, and no endoleak was found ( Figure 4F).

### Case 5

**Figure 5.**
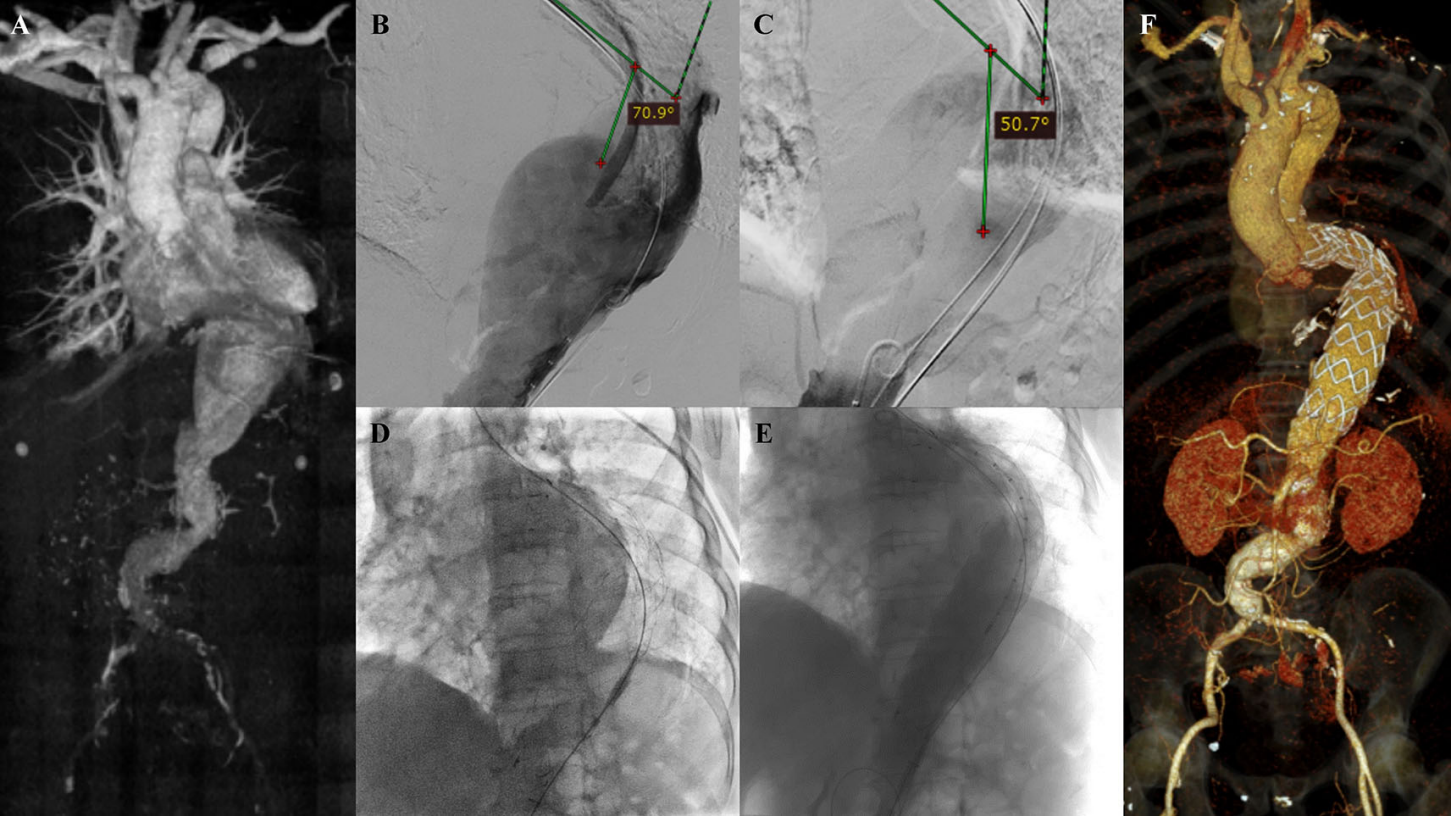
Thoracic endovacular aortic repair with multiple stiff wire technique of the 5 ^th^ patient. A. Preoperative volume rendering computed tomography angiography (CTA); B. Initial aortography with two stiff wires revealed 70.9° angulation of the descending thoracic aorta; C. Additional stiff wire was added and straighten the angulation into 50.7°; D. Stent graft deployment; E. Final aortography; F. Follow up volume rendering CTA after three months.

A 63-year-old man presented to our hospital with tearing chest pain that radiate to his back in the past 3 days. The patient had a history of hypertension and he was a smoker. CTA revealed an aortic aneurysm with extensive mural thrombus with aorta descendent’s maximum diameter of 53.5 mm, thoracoabdominal aorta maximum diameter of 92.5 mm, suprarenal aorta maximum diameter of 98.1 mm, infrarenal aorta maximum diameter of 51.8 mm, with angulation of 70.2°, and suspected rupture at peritoneal and thorax cavity ( Figure 5A). The patient was than prepared for thoracic endovascular aortic repair (TEVAR) procedure.

The TEVAR procedure was done with puncture from right and left femoral access. The MSW technique was applied to this patient. Initial aortography revealed an aortic aneurysm at descending thoracic aorta until abdominal aorta with 70.9° angulation of the descending thoracic aorta ( Figure 5B). Additional stiff wire was added with a total of three stiff wires used to straighten the aorta. The angulation was then reduced into 50.7° ( Figure 5C). SEAL Thoracic Stent Graft 36 × 200 mm (S&G Biotech, Yongin, Korea) was deployed at descending thoracic aorta ( Figure 5D). Evaluation aortography revealed good stent position but there was type IV endoleak. Observation for 10 minutes was done, and another evaluation aortography revealed no more endoleak ( Figure 5E). The TEVAR procedure was done without any complications. The total contras used was 275 mL Iohexol 755 mg/mL, DAP 179.63 Gy.cm ^2^, and fluoro time was 43.10 minutes.

The patient was discharged without any complaint. Follow-up CTA after 3 months revealed stent expansion of 18.2 mm at aorta descendent (maximum diameter with thrombus was 56 mm), stent expansion of 29.4 mm at thoracoabdominal aorta (maximum diameter with thrombus was 88.2 mm), no stent migration, and no endoleak was found ( Figure 5F).

### Case 6

**Figure 6.**
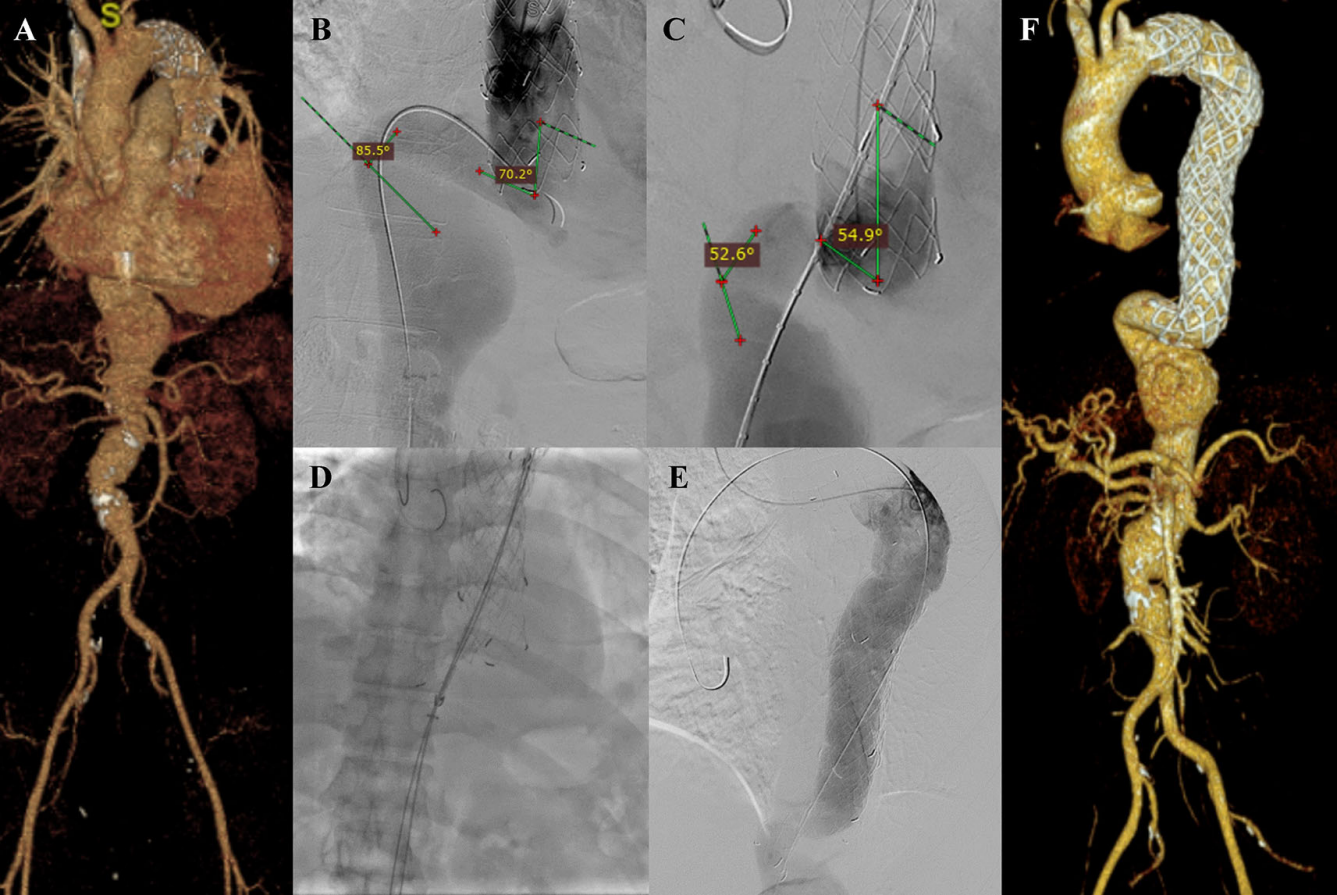
Thoracic endovacular aortic repair with multiple stiff wire technique of the 6 ^th^ patient. A. Preoperative volume rendering computed tomography angiography (CTA); B. Initial aortography showed 70.2° and 85.5° angulation of the descending thoracic aorta; C. Two stiff wire was added and straighten the angulation into 54.9° and 52.6°; D. Stent graft deployment; E. Final aortography; F. Follow up volume rendering CTA after three months.

A 45-year-old man presented to our hospital with lower abdominal pain radiating to epigastric in the past 3 months that getting worse in the past 3 days. The patient had undergone TEVAR with indication of Stanford A acute aortic dissection 2 years earlier. CTA revealed an impending rupture of pseudoaneurysm sized ±7 × 12 cm with leakage from the distal part of the previous TEVAR stent with 72° and 85.4° angulation of the aorta, distal from the previous stent ( Figure 6A). The patient was then prepared for an extension TEVAR procedure.

The procedure was done with puncture from right brachial, right and left femoral access. Initial aortography revealed dissection of the descending thoracic aorta with an entry tear from the distal of the previous TEVAR stent. There were 70.2° and 85.5° angulations of the descending thoracic aorta ( Figure 6B). Wire snaring was done from the right brachial artery to the right femoral artery. Wire exchange was done with the 0.035″ × 300 cm Lunderquist Extra Stiff Wire (Cook Aortic Interventions, Bloomington, USA) and additional extra stiff wire was inserted from the left femoral artery. The angulations were straightened into 54.9° and 52.6° ( Figure 6C). SEAL Thoracic Stent Graft 36(32) × 130 mm (S&G Biotech, Yongin, Korea) was deployed at descending thoracic aorta until abdominal aorta, overlapped with the previous stent ( Figure 6D). Evaluation aortography revealed good stent position without any endoleak ( Figure 6E). The TEVAR procedure was done without any complications.

The patient was discharged without any complaint. Follow-up CTA after 3 months revealed stent expansion of 30.8 mm at aorta descendent (maximum diameter with thrombus was 98.7 mm), stent expansion of 33.9 mm at thoracoabdominal aorta (maximum diameter with thrombus was 114.3 mm), no stent migration, and no endoleak was found. There was an abdominal aortic aneurysm sized 47.33 mm at supra renal and 43.5 mm at infrarenal ( Figure 6F). The patient didn’t have any complaints. The total contrast used was 80 mL Iodixanol 642 mg/mL, DAP 196.73 Gy.cm ^2^, and fluoro time was 43.09 minutes.

## Discussion

The prevalence of severe neck angulation in EVAR is noteworthy. A single-center study in Greece by Karathanos *et al.* found that 34 of 317 EVAR patients (10.7%) had a neck angle >60°. ^9^ EVAR with severe neck angulation compared to non-severe neck angulation was associated with a significantly higher rate of type 1a endoleak until 3 years (5.6% vs 2.6%; p<0.00001; OR 2.57 95% CI 1.62–4.07), neck-related secondary procedure until 3 years (13.1% vs 9%; p<0.05; OR 1.42 95% CI 1.04–1.96), migration rates until 1 year (5.4% vs 4.0%; p<0.05; OR 1.41 95% CI 1.03–1.94), aneurysm-related and all-cause mortality until 1 year (6.4% vs. 4.3%; p<0.05; OR 1.51 95% CI 1.16–1.98), but not related to aneurysm sac increase and rupture. ^10^ High-tortuosity neck in TEVAR also has its problem related to a significantly higher proportion of endoleaks, a potential risk factor for proximal stent-graft collapse or infolding, renal failure, stroke, longer operation time, and lower survival rate. ^11^^,^^12^ Another problem in EVAR practice is the tortuosity of the iliac arteries which is associated with a significantly greater rate of limb occlusion (53% vs 12% P<0.03). Additional stenting is well accepted to address that complication. ^13^ However, kinking of the iliac limbs can also be an issue. ^14^

Data from the ANCHOR trial showed that the use of Aptus Heli-FX EndoAnchor System (Medtronic, Santa Rosa, CA, USA) was associated with higher but not significant 2 years rate of freedom from type Ia endoleak, neck dilation, and sac enlargement compared to the control group. ^15^ The Kilt technique appeared to significantly straighten neck angle without leaving additional death or re-intervention during 22 ± 15 months follow up and no newly developed endoleaks during 15.9 ± 16.4 months follow up. ^16^ Although those techniques showed a good result in overcoming the angulation of the aortic neck, they add additional complexity to the procedure, specialized devices, need special training for the operator, and also greater cost of the procedure. Several proposed simple novel techniques such as directional tip control technique, push-up technique, and using a large bore sheath to untwist tortuous iliac arteries seem promising but need further investigation. ^5^^,^^17^^,^^18^

Our MSW technique provides a simple solution to manage neck/artery angulation. It can be used in the thoracic aorta, thoracoabdominal aorta, abdominal aorta, or iliac artery. Our experience applying the technique in six different scenarios showed satisfactory results. There is no type I endoleak, stent migration, or enlargement of the aneurysm size found after follow-up CTA in all but one of our patients. The determination of how many stiff wires would be used in the procedure is based on operator judgment until the tortuosity of the vessel is straightened enough to facilitate stent graft deployment. It is important to always cover the stiff wire with a catheter, so the stiff wire doesn’t contact directly with the vessel and the risk of dissection is reduced. We also recommend oversizing 20% of the main body stent graft to create an optimal seal between the stent graft and the aortic wall.

Further studies will be made for the MSW technique with a larger population and study design with a longer follow-up period. Clear indications should be made with the straightening degree calculation of the technique.

## Conclusion

MSW technique is a simple and effective technique to modify aortic neck angulation/iliac artery tortuosity in TEVAR or EVAR procedure. The technique is safe and reproducible although further research with larger sample sizes is needed to validate the results.

## Consent

Written informed consent has been obtained from the patients for publication of the case and accompanying images.

## Data Availability

All data underlying the results are available as part of the article and no additional source data are required. Figshare. CARE checklist for ‘Endovascular management of aortic aneurysm with severe neck angulation and/or iliac artery tortuosity using multiple stiff wire technique: a case series’. DOI: https://dx.doi.org/10.6084/m9.figshare.23925234. Data are available under the terms of the Creative Commons Zero “No rights reserved” data waiver (CC0 1.0 Public domain dedication).
