# Supplementary material for: Endovascular management of aortic aneurysm with severe neck angulation and/or iliac artery tortuosity using multiple stiff wire technique: a case series
Source: F1000Res. 2024 Jan 5;12:1137. Originally published 2023 Sep 12. [Version 2] doi: 10.12688/f1000research.140435.2 (PMC10905129; doi:10.12688/f1000research.140435.2)
Supplement: Peer review history

# Reviewer report: Reviewer response for version 2

**DOI:** 10.5256/f1000research.160558.r235884

**Published:** 2024-02-20

**Authors:** Apostolos G. Pitoulias

**Affiliations:** 1 Aristotle University of Thessaloniki, Thessaloniki, Greece

Authors revised their article according to suggestions.

 They provided adequate information about hostile neck, as well as more information about the angulation of the neck and intraoperative data.

 In conclusion the revised manuscript is approved. No further comments are deemed necessary.

Are enough details provided of any physical examination and diagnostic tests, treatment given and outcomes?

Yes

Is the background of the cases’ history and progression described in sufficient detail?

Yes

Is sufficient discussion included of the importance of the findings and their relevance to future understanding of disease processes, diagnosis or treatment?

Yes

Is the conclusion balanced and justified on the basis of the findings?

Partly

Reviewer Expertise:

vascular surgery

I confirm that I have read this submission and believe that I have an appropriate level of expertise to confirm that it is of an acceptable scientific standard.

# Reviewer report: Reviewer response for version 1

**DOI:** 10.5256/f1000research.153785.r221046

**Published:** 2023-11-10

**Authors:** Johannes Nugroho eko putranto

**Affiliations:** 1 Department Cardiology and Vascular Medicine, Faculty of Medicine, Airlangga University, Surabaya, East Java, Indonesia

The article titled "Endovascular Management of Aortic Aneurysm with Severe Neck Angulation and/or Iliac Artery Tortuosity Using Multiple Stiff Wire Technique: A Case Series" discusses a novel technique for addressing aortic neck angulation and iliac artery tortuosity in the context of endovascular aortic repair. The case series presents outcomes for six patients who underwent the procedure. The manuscript is well-structured, and the information provided is clear and detailed. However, there are some feedback.

 It provides a comprehensive background on the importance of a suitable aortic neck in endovascular aortic repair and the associated challenges. The references cited are recent and relevant. The authors correctly acknowledge that existing techniques, such as the Kilt technique and EndoAnchor System, have shown promise in managing angulated aortic necks but also add complexity and cost to the procedures. The MSW technique is presented as a simpler and cost-effective alternative, which is supported by the successful outcomes in the cases presented. 

 However, it would be beneficial to include specific statistics or data on the prevalence of aortic neck angulation and its clinical impact to underscore the significance of the problem. In the conclusion , it should reiterate the need for further research and larger sample sizes to validate the results.

 The study could benefit from further elaboration and comparison of the MSW technique with other methods, emphasizing its potential advantages and limitations. It would also be helpful to discuss the long-term implication using the MSW technique.

 In summary, the case reports and the discussion section collectively highlight the potential of the MSW technique in addressing aortic neck/artery angulation. The cases demonstrate its effectiveness, and the discussion section provides context and identifies the need for further research. The article continues to show promise, future studies and a more comprehensive discussion of the technique's advantages and limitations are necessary to strengthen its impact.

Are enough details provided of any physical examination and diagnostic tests, treatment given and outcomes?

Yes

Is the background of the cases’ history and progression described in sufficient detail?

Partly

Is sufficient discussion included of the importance of the findings and their relevance to future understanding of disease processes, diagnosis or treatment?

Yes

Is the conclusion balanced and justified on the basis of the findings?

Yes

Reviewer Expertise:

Vascular

I confirm that I have read this submission and believe that I have an appropriate level of expertise to confirm that it is of an acceptable scientific standard.

# Reviewer report: Reviewer response for version 1

**DOI:** 10.5256/f1000research.153785.r210037

**Published:** 2023-10-12

**Authors:** Apostolos G. Pitoulias

**Affiliations:** 1 Aristotle University of Thessaloniki, Thessaloniki, Greece

This is a quite interesting manuscript considering a simple and safe solution for the treatment of AAA with hostile neck anatomy and tortuous iliac arteries.

 Considering the introduction, more information about the other aspects of hostile neck (reverse tapering, width, circumferential thrombus or calcification and length) should be included.

 Considering the case presentations it would be great if you could also include intraoperative angulation measurements before and after the placement of the superstiff wires, in order to better evaluate the changes in the angulation of the neck. The same could also be applied for the iliac arteries.

 Also some more intraoperative data, such as operation-time, contrast agent volume and experience of the vascular surgeon, should be documented to better evaluate the safety, reproducibility, simplicity and effectiveness of the msw technique, especially since it is stated in the introduction and discussion that the kilt and endostapling techniques, although efficient and safe add more devices and complexity to the procedure. 

 Considering the discussion, it is lacking information about the tortuosity of the iliac arteries (although it is stated in the tile of the manuscript.)

 Considering the Conclusion, the safety and reproducibility of the technique should be also included. Also here more information about the iliac tortuosity are required. 

 Finally major linguistic revision is required.

Are enough details provided of any physical examination and diagnostic tests, treatment given and outcomes?

Yes

Is the background of the cases’ history and progression described in sufficient detail?

Yes

Is sufficient discussion included of the importance of the findings and their relevance to future understanding of disease processes, diagnosis or treatment?

Yes

Is the conclusion balanced and justified on the basis of the findings?

Partly

Reviewer Expertise:

vascular surgery

I confirm that I have read this submission and believe that I have an appropriate level of expertise to confirm that it is of an acceptable scientific standard, however I have significant reservations, as outlined above.
